# Homœopathia.—Anecdote

**Published:** 1850-10

**Authors:** 


*Homœopathia.—Anecdote.*—We copy the following from a communication in the New York Medical Gazette:—

We conclude our notice of Homœopathy by relating an incident of sober truth, which not long since is said to have occurred at the Springs, and only withhold the name of our informant, and of the watering place, that the parties may not be recognized. A merchant in a neighboring city, whose business brought him in contact with the article called "Pearl Barley," filled his vest pocket with a handful of the grains when about to leave home, and amused himself by chewing them on his journey. On his arrival at his hotel at the Springs, he found a number of invalid acquaintances, who were wont to discourse on the merits of physicians, one of whom was a sturdy believer in Homœopathia, and loud in praises of the little pellets of sugar of milk. Taking from his pocket a grain of the Pearl Barley, he placed it upon his tongue in the presence of the company, and united his testimony to the value of these pellets, which the very fine grains he used closely resembled; he, moreover, informed them that he carried these treasures in his pocket, and could spare them to any who might have occasion to try Homœopathy. Very soon one and another of these invalids, ladies and gentlemen, availed themselves of the courteous proposition, and applied to the merchant for one of his little pills, to each of whom he gave a grain of Pearl Barley, accompanied by an eulogy upon Homœopathy. In every instance the patients were relieved, and in some cases the effects were wonderful; so that the astonishing virtues of the little pills became the theme of continual conversation, and converts were as numerous as patients, so that the stock in the gentleman's pocket was soon exhausted. But at length the merchant broke the charm by announcing at the public table, in the presence of these restored invalids, that he had only humbugged all parties; that his little pills were only fine grains of Pearl Barley, of which a bushel might be had for a less number of shillings than it would take dollars to fee a Homœopathist. The result of this disclosure was most disastrous, like that of Dr. Haygarth's substituting painted wooden tractors for Perkins' magnetic metallic ones; for the patients forthwith relapsed by the return of all their maladies, and before sundown the Homæpathic cures exploded.

